# Trends in Anterior Cruciate Ligament Injury and Recovery in Professional Snowboarders: The Extreme Sport of Snowboardcross

**DOI:** 10.7759/cureus.50683

**Published:** 2023-12-17

**Authors:** Hannah R Popper, Patrick F Szukics, Jenna Feldman, Elizabeth Ford, Manuel Pontes, Sean McMillan

**Affiliations:** 1 Orthopaedic Surgery, Jefferson Health, Stratford, USA; 2 Orthopaedic Surgery, Inspira Health Network, Vineland, USA; 3 Marketing, Rowan University, Glassboro, USA; 4 Orthopedic Surgery, Virtua Health, Cherry Hill, USA

**Keywords:** acl reconstruction, anterior cruciate ligament, sports related knee injuries, winter sports, snowboarding

## Abstract

Introduction

An anterior cruciate ligament (ACL) tear is a devastating injury for athletes that is predominantly low energy and non-contact in nature. ACL tears are one of the most well-researched injuries in sports, however, scant research has been done on competitive snowboarders. *Boardercross* is a relatively new sport introduced to the Winter Olympics in 2006. Initially, it entailed four snowboarders racing head-to-head down a course of obstacles in a race to the finish, with the top two riders advancing to the next rounds. It has since expanded to six racers traveling up to 60 mph and jumps up to 100 feet in length in a head-to-head race to the finish. This extreme sport puts its athletes at risk for serious injury, requiring investigation.

Purpose

Investigate the prevalence of ACL tears in the extreme sport of boardercross, evaluate sport-specific factors that may put athletes at higher risk, and report return to sport data.

Methods

An expedited IRB approval was obtained. A survey was distributed to athletes via e-mail to national/regional coaches of countries with competitive boardercross teams. Professional coaches distributed the survey and secondarily distributed it to athletes.

Results

Sixty-six competitive snowboardcross athletes responded to the email surveys 48.5% of respondents had torn their ACL at least once in their career. Of the female respondents, 55.6% suffered at least one ACL tear, and 43.6% of male respondents suffered at least one ACL tear. 31.2% suffered more than one ACL tear during their career. Of those who tore their ACL, 91.3% (p <0.001) tore their front leg. 100.0% of the respondent athletes returned to sport post-ACL reconstruction.

Conclusion

Professional boardercross racers are at a higher risk of tearing their ACL than other winter sport athletes, including alpine skiers. A predominance of ACL injuries occurred on the front leg during landing from an aerial maneuver. All respondent athletes returned to the sport after injury, with approximately half returning within six months. Although no statistical significance was achieved, the data provided trends on risk factors related to ACL injuries among snowboardcross athletes.

## Introduction

Anterior cruciate ligament (ACL) injuries are one of the most documented and studied in orthopedic and sports medicine. A recent study 2019 reported an incidence of ACL tears to be 68.8 per 100,000 people [1]. While much has been written on ACL injuries and return to play (RTP) in team sports, before a case series from 2013, there was no reported literature on RTP after ACL among snowboard athletes. This case series revealed a 70% RTP rate among X-Games snowboarders [2]. 

A 2016 report estimated there are approximately 117 million skiers worldwide compared to 7.7 million snowboarders, of whom 82% were under 34 years [3]. ACL injuries among alpine skiers have been studied despite there being a paucity of data on these injuries in their snowboarding counterparts [4-6]. In a retrospective review of the rate of ACL injuries at mountain lodges, there was an incidence of 17% ACL tears among alpine skiers compared to an incidence of 1.7% ACL injuries among snowboarders [5]. Another study found a similar discrepancy between these two groups, reporting overall knee injuries of 17% in snowboarders versus 39% in alpine skiers [7].

Several factors account for the discrepancy in the number of ACL injuries seen between skiers and snowboarders, including foot positioning and the construct of fixation of the lower extremity to the ski or snowboard. Snowboarders stand sideways on their boards, similar to a surfer’s position. The rear foot is approximately 90 degrees to the long axis of the board, and the front foot is between 60 and 90 degrees to the long axis [8]. Turning is performed by shifting body weight to engage the board's edge underneath the front foot, allowing the board's camber to flex and dig the rest of the edge into the snow. To accommodate for the differences between skiing and snowboarding in stance and mode of maneuvering, the boot, board, and bindings were modified for snowboarding [8]. During the early years of snowboarding's popularity, soft-shelled boots and the absence of release bindings were used, contributing to the high rate of ankle injuries among snowboarders compared to skiers [9]. To correct for this, hard-shelled boots and soft boots with rigid ankle support inserts were favored to reduce the risk of ankle injuries. In a case series of 415 injured snowboarders, there was a higher rate of ankle injuries among snowboarders using soft boots, while there was a higher rate of knee injuries among those who used hard-shelled boots [9,10].

Since introducing two snowboarding events at the 1998 Winter Olympic Games, the sport has grown on an international stage to seven Olympic events in 2022. Snowboardcross, a relatively new sport, made its Olympic debut during the 2006 Winter Olympic Games in Torino, Italy. At the 2022 Winter Olympic Games, there were 238 snowboard athletes, of which 64 qualified for snowboardcross events [11]. Snowboardcross is a physically demanding sport involving competing on courses in which they travel up to 60 miles per hour and completing jumps over 80 feet in length and 30 feet in height. This, in addition to having their feet strapped into fixed-angle bindings, can make snowboardcross athletes particularly susceptible to knee injuries. Within the professional snowboarding community, the rate of ACL tears is believed to be significantly higher than previously reported, partly due to the high-impact nature of competitive snowboarding. In a study comparing injuries in recreational versus competitive snowboarders, athletes in snowboardcross and freestyle suffered more injuries to the lower extremities than their other competitive counterparts. Of all five disciplines, Snowboardcross had the maximum number of days missed per injury [12]. In that same study, almost half of all boardercross athletes surveyed reported that their injuries impaired their daily lives [12]. Both factors support the level of danger implicated in the sport of boardercross.

This paper examines ACL injury trends and returns to sport in professional snowboardcross athletes. Examining factors potentially predisposing this population to an unusually high rate of injury will be discussed. Despite the small population, we intend to highlight trends within the competitive snowboarding community and examine return-to-play (RTP) rates within this cohort.

## Materials and methods

**Figure 1:**
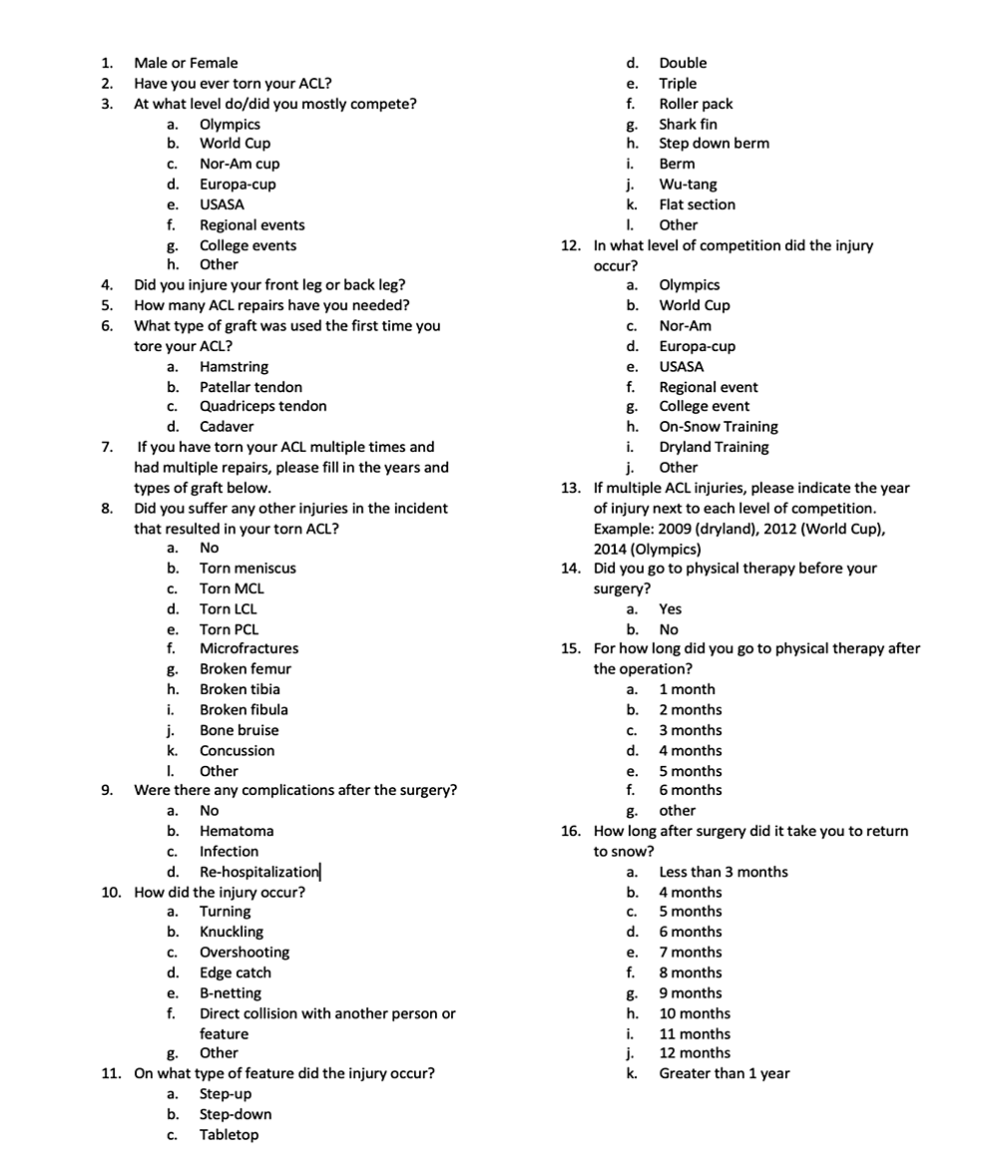
Survey completed by athlete participants If athletes did not tear their ACL, the survey was not completed past question 2; if an athlete tore his or her ACL, all 16 questions were answered.

Expedited IRB approval was granted for this study. The Rowan School Of Osteopathic Medicine Ethics Committee reviewed the study. Once approval was granted, professional snowboard coaches, who were named from each country for the professional and developmental teams, were contacted via e-mail to explain the study and ask for help disseminating the surveys to their athletes. During the surveys, 120 athletes worldwide were registered as active snowboardcross competitors with recognized organizations, including the Federal International de Ski (FIS) and the USA Snowboard Association (USASA). The survey was also accessible online, where athletes were directed to an informed consent page. All surveys were anonymous. After agreeing to the consent, they were directed to the survey (Figure 1). The lead researcher inter-IRB team developed the questions in-house to capture data pertinent to the study's purpose. Answers to questions were a combination of multiple choice and free response. For athletes who had torn their ACL, the full 16-question survey was completed. For athletes who had not torn their ACL, only the first two questions of the survey were completed. Responses to the surgery were collected via Survey Monkey. The request to complete the surveys was sent twice to the coaches for dissemination.

Statistical analysis

For this research, statistical analyses were performed using R software version 4.3.1. The R package, ratesci, was used to estimate proportions, and risk differences, calculated by differences between proportions and confidence intervals [13]. For the analyses reported in this paper, the scoreci function in the ratesci package was used. The scoreci function uses the Wilson method to estimate confidence intervals (CIs) for proportions and the Newcombe method (based on the Wilson score) to estimate CIs for differences in proportions [14,15].

## Results

Sixty-six responses were recorded by the close of the survey. At the time of the survey emailing, 120 individuals were listed as meeting criteria worldwide as registered competitive snowboardcross athletes. It is unknown, however, if all 120 athletes received the surveys due to dissemination of the surveys through the registered coaches. Of the 66 athletes who responded to the survey, 39 were male, and 27 were female. 48.5% (32/66) of respondents had suffered an ACL tear while snowboarding. Of the 39 males, 43.6% (17/39) suffered one or more ACL tears, compared to a reported rate among female respondents of 55.6% (15/27); p=0.343. Of the 66 athletes who participated in the survey, 48 (72.7%) competed at the World Cup and/or Olympics. 15 (22.7%) of the athletes who responded reached the Nor-Am or Europa Cup level of competition, and 3 (4.5%) athletes reached the regional level of competition (Table 1).

**Table 1:** Relationship between gender and highest level of competition for ACL tears Abbreviation: RD, risk difference; N, Number of patients, n, % of patients with ACL tears; ACL: anterior cruciate ligament ^1 ^Male gender was used as a reference, and ^2 ^Regional events when comparing risk differences for ACL tears when separating participants by gender and highest level of competition, respectively. Note: Risk difference is calculated by the difference between proportions using the above as references.

		Patients (N)	ACL Tears - % (n)	RD [95% CI]	Chi-Square	p
Gender	Female	27	55.6% (15)	12.0% [-12.5%, 35.1%]	0.9	0.343
Male (Ref)^1^	39	43.6% (17)				
Highest Level of Competition	World Cup/Olympics	48	47.9% (23)	-65.6% {-52.1%, 6.5%]	3	0.083
Nor-Am Cup/Europa Cup	15	40% (6)	-60% [-80.6%, 3.7%]	3.4	0.065	
Regional Events (Ref)^2^	3	100% (3)				

Of 27 female respondents, 20 (74.1%) participated in the World Cup, and 18 (66.7%) participated in the Olympics. Out of 39 male respondents to the survey, 23 (59.0%) participated in the World Cup, and 12(30.8%) participated in the Olympics. Female respondents were significantly more likely to have participated in the Olympics (66.7%) than male respondents (30.8%) (risk difference =35.9%, 95%CI= [11.4, 56.2]). Of those who tore their ACL, 21 competed primarily at the World Cup level, and a total of 15 competed at the Winter Olympics. 6 (40%) of those who competed at the Nor-Am or Europa Cup at the highest level suffered one or more ACL tears (p=0.065). 

Out of the 32 athletes who suffered more an ACL tear, most athletes, 90.6% (29), had an ACL tear on their front leg (p<0.001), while only 9.4% (3) had an ACL tear on their back leg (Table 2). Several athletes suffered multiple ACL tears. Eight athletes required two reconstructions, while two athletes required a total of four reconstructions.

**Table 2:** Rate of ACL Tears of Athlete’s Front vs. Back Leg Abbreviation: CI, Confidence Interval; n, number of ACL tears/athletes injured per leg; N, total number of ACL tears among athletes/total number of athletes with ACL tears

	n	N	% [95% CI]	Chi-Squared	p-value
Back Leg (Tears)	4	46	8.7% [3.4%, 20.3%]	31.39	< 0.001
Back Legs (Athletes)	3	32	9.4% [3.2%, 24.2%]	21.13	< 0.001
Front Leg (Tears	42	46	91.3% [79.7%, 96.6%]	31.39	< 0.001
Front Leg (Athletes)	29	32	90.6% [75.8%, 96.8%]	21.13	< 0.001

In addition to the leg that was injured, the mechanism of injury was also reported among athletes. This included knuckling/undershooting the desired target, overshooting, landing, turning, collision, or another mechanism. The mechanism was reported for 37 ACL tears among the athletes. Knuckling and overshooting were most commonly associated with ACL tears among our cohort of athletes (Table 3).

**Table 3:** Injury Mechanism Abbreviation: CI, confidence interval; N, number of tears per mechanism Knuckling = landing on the transition hump between the gap and landing area- the rider came up short of the inclined landing zone. Overshoot = the rider jumped farther than the inclined landing zone, falling from a greater height than intended and landing on flatter terrain. Landing = the rider landed on the designated inclined landing zone.

	N	% [95% CI]
Knuckling	18	48.6% [33.4%, 64.1%]
Overshoot	12	32.4% [19.6%, 48.5%]
Landing	2	5.4% [1.5%, 17.7%]
Turning	2	5.4% [1.5%, 17.7%]
Collision	1	2.7% [0.4%, 13.8%]
Other	2	5.4% [1.5%, 17.7%]
Total	37	

Hamstring autograft was the primary graft choice, 43.5% (20/46 ACL reconstructions). Allografts (cadaver grafts) were the next most common graft choice (13/46). Other autologous grafts used were patellar tendon (12/46) and, less commonly, quadriceps tendon (1/46) (Table 4). Although lacking statistical significance, athletes who received a cadaver graft for primary reconstruction suffered a higher rate of re-injury compared to those when an autologous graft was used, with a risk difference of 21.9% (p = 0.150) (Table 5). Of note, one female respondent reported 4 ACL reconstructions to the same knee over seven years. This patient reported two hamstring autografts, followed by two cadaver allografts. A second female respondent noted four ACL reconstructions to the same knee over six years with the utilization of allograft for the initial two procedures.

**Table 4:** Relationship between type of ACL Graft and ACL-Reinjury Rate Abbreviations: CI, confidence interval; H, hamstring; PT, patellar tendon; QT, quadriceps tendon; N, number of ACL injuries treated with each graft type; n, number of re-injuries with each graft type

	Grafts	ACL Reinjury	
	N	n	% [95% CI]
Cadaver	13	6	46.2% [23.2%, 70.9%]
Non-Cadaver (Autologous)	33	8	24.2% [12.8%, 41.0%]
Hamstring (H)	20	6	30.0% [14.5%, 51.9%]
Patellar tendon (PT)	12	2	16.7% [4.7%, 44.8%]
Quadriceps tendon (QT)	1	0	0.0% [0.0%, 79.3%]

**Table 5:** Graft Failure Rates – Cadaver versus Autologous Abbreviations: RD, risk difference; CI, confidence interval.

Grafts		ACL Injury RD [95% CI]	Chi-Squared	p
Cadaver	Autologous	21.9% [-7.2%, 50.6%]	2.07	0.150

Out of 32 patients with ACL tear, 23 (71.9%) had concomitant injuries, and 28.1% (9) had no concomitant injuries. 62.5% (20) reported meniscus injury, 31.3% (10) reported bone bruise, 9.4% (3) reported torn MCL(medial collateral ligament), 2 (6.3%) torn LCL(lateral collateral ligament), 0% torn PCL(posterior cruciate ligament). One respondent suffered a tibial plateau fracture, and one reported a ruptured patellar tendon. 81.3% (26) experienced no complications postoperatively. 6.3% (2) reported a hematoma formation, 9.4% (3) reported infection, 1 required arthroscopic scar tissue removal, 1 required manipulation under anesthesia, and 1 experienced prolonged hamstring pain.

## Discussion

Snowboarding is a rapidly growing sport, particularly among the younger population. Although snowboarding is less popular than traditional impact sports, its acceptance has grown to the stature of having multiple Olympic events. Nevertheless, there is a relative lack of literature on ACL injuries within the sport, particularly the high-speed subset of snowboardcross. These authors aimed to examine the risk of ACL injuries among competitive snowboardcross athletes.

ACL tears in our cohort were found to be high (48.5%) compared to ACL injury rates among alpine skiers within the literature (15-21%) [5,16]. Studies have shown that the disparity of ACL tears among men and women can be sport-specific [17,18]. Some studies regarding gendered ACL tears show that female skiers are two to six times more likely to tear their ACL than their male counterparts [19,20]. Another study attempted better control of male vs female skiers and discovered the incidence was similar in controlled professional ski instructors and patrollers at a single resort [7]. In the present study, gender differences were not statistically significant (p= 0.343) between male and female snowboardcross competitors who tore their ACL, however, the sample size may be a limiting factor. 

This is the largest cohort examined in the literature on ACL tears and competitive snowboarders. In our study, 100% of respondents who tore their ACL returned to sport, although it is unknown if they qualified for their previous level of competition. 48.3% returned to competition within six months of the injury, and 89.7% of respondents returned to sport within nine months of reconstruction. In a previous study from 2013 that investigated the return to sport in X-Games skiers and snowboarders who tore their ACL; they found that the return to sport was 70.0% among snowboarders (7/10). A 4.0% re-tear rate (1/25) (2) was also noted. This compares to our study, which found a 31.3% re-tear rate. Another study found a mean return to skiing in 5.4 months after ACL reconstruction, similar to our study findings in snowboarders [21]. Table 6 compares return to play and subsequent re-tear rates from our respondents versus a similar cohort within the literature. The athletes in our study who tore their ACLs demonstrated a significantly greater return to sport; however, they also had higher graft failure rates than data found in the literature. Both the high return to play and the re-tear rate may be explained by the high level of competition that the majority of study participants competed in.

**Table 6:** Return to play and re-tear rates compared to previous literature Abbreviations: n = number of athletes returned to play/number of athletes with re-tears, N = Number of total athletes Note 1: The study percentages of return to play (100%) and retear were higher than the literature. Literature values represent 70% of return to play and 4% re-tear rate [2]. Note 2: 95% Confidence Lower Limits were greater than literature value* (Study values significantly greater than literature). *Compared to values from Erikson et al. 2013 [2].

	Study Values			Literature
	N	n	% [95% CI]	
Return to Play	32	32	100.0% [89.3%, 100.0%]	70 %
Re-tear	32	10	31.3% [18.0%, 48.6%]	4 %

Due to the high rate of ACL tears among snowboardcross athletes, it is important to examine the risk factors contributing to susceptibility to these injuries. The mechanism of ACL tears in snowboarders differs from skiers [22-24]. In snowboarders, maximal eccentric quadriceps contraction at the time of landing may lead to internal rotation of the front knee, predisposing it to injury [25]. On the contrary, when a skier lands in slight flexion, quadriceps loading produces significant anterior tibial translation and internal rotation, resulting in ACL rupture [26]. Additionally, the positioning of the feet on the snowboard and the construct fixation of the feet to the board may contribute to the increased risk of injury.

**Figure 2:**
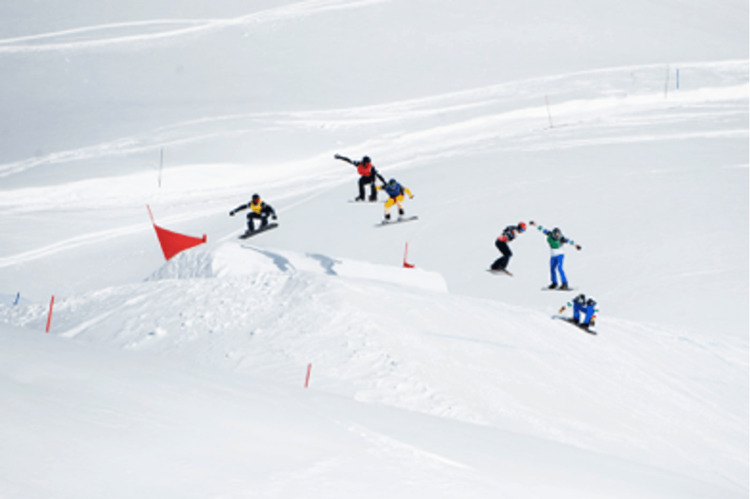
Athlete positions during boardercross runs Riders in yellow, red, and blue bibs are in mid-air in an aerodynamic position with flexed hips and knees. Riders in black and green bibs are preparing for landing by extending their hips and knees to absorb the force of landing- this semi-flexed position allows quadriceps loading, anterior tibial translation, and internal rotation of the leg, which puts riders at risk for ACL tear. The rider in the lead in the white bib has returned to the max aerodynamic position after a successful landing. Photo courtesy of FIS.

**Figure 3:**
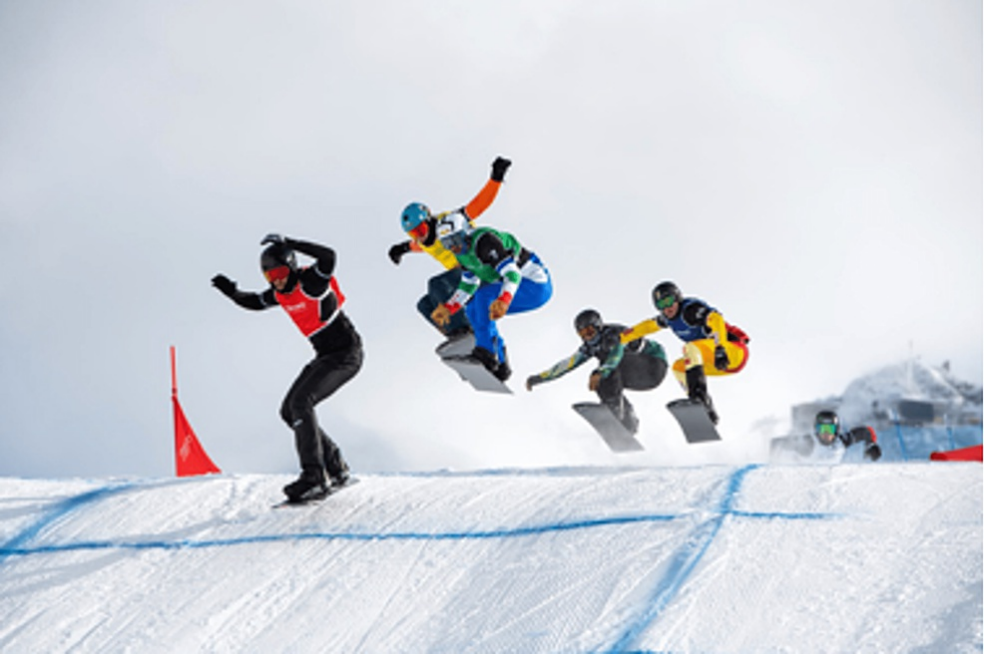
Athlete position during landing The rider in the red bib represents the semi-flexed position naturally encountered by the rider when landing. Photo courtesy of FIS.

To improve aerodynamics during jumping, boardercross athletes are in a tucked position with flexed knees and hips (Figure 2). In anticipation of landing, the knees and hips extend until touchdown, and the body returns to the flexed position. At the time of impact, the knees are only slightly flexed, which is the position of greatest stretch on the ACL [26-28]. Looking at ACL strain during dynamic jump landing in snowboarders, it was found that in anticipation of landing, the knee goes from a flexed to semi-extended position, followed by rapid flexion after landing [28]. Similarly, multiple studies have shown the semi-extended position to be the most susceptible position during jump landing for ACL tears to occur [29,30]. Figure 3 demonstrates an example of this knee position during landing. Errors in landing from an aerial maneuver can alter appropriate knee kinematics during impact, as was reported in our data. Understanding the propensity for athletes to injure themselves in these ways may lead snowboardcross coaches and trainers to alter and strategize strengthening regimens to better prepare athletes for landings.

Limitations do exist in our study. The relatively small sample size of 66 respondents does impair statistical significance; however, to date, this is still the largest cohort of ACL injuries studied in competitive snowboarders and snowboardcross athletes. It is unknown if all 120 registered competitive snowboardcross athletes received the survey when it was sent out as they were disseminated through a third party without the confirmation of receipt, therefore response rate was unable to be calculated. Additionally, the surveys were sent out in English; however, given that some athletes are international, a language barrier may have been an impediment. Additionally, the return to competition of the athletes post-ACL reconstruction did not specify if they returned to the same level of pre-injury competition. Furthermore, the data may be skewed given two athletes had 4 ACL surgeries, and their recoveries and re-injury timelines varied with each surgery. Regardless of these limitations, this study provides relevant information on risk factors related to ACL injuries among snowboardcross athletes that can help guide training and competition preparation techniques to help decrease injury rates.

## Conclusions

Though this study failed to achieve statistical significance secondary to the small sample size in most trends measured during this study, there were important trends that can contribute to a greater understanding of ACL tears in snowboardcross. ACL tears occurred almost exclusively in landing from an airborne obstacle, and the front knee sustained almost all of the ACL tears (p< 0.001). A majority of injuries occurred in a competition setting rather than in training. Though there are more men in the competitive field, there was a near-equivalent rate of rupture between males and females (p = 0.343). These injuries significantly impact the days missed from the competition, given the timelines of recovery reported. 100% of respondent athletes who tore their ACL did return to competition, with half returning within six months, which is significantly higher than reported in previous literature. Further examination of this growing field of athletes and injuries to the ACL is warranted to help formulate algorithms to decrease rates of injury potentially.
